# Prevalence and Risk Factors of Cervical Lesion Among Married Women With Low Socioeconomic Status: A Study Based on a Cervical Cancer Screening Program

**DOI:** 10.3389/ijph.2025.1608482

**Published:** 2025-06-20

**Authors:** Mingyu Zhang, Haoyue Wang, Ruoxi Ding, Wen Li, Ping He, Hui Li

**Affiliations:** ^1^ Centre for Health Management and Policy Research, School of Public Health, Cheeloo College of Medicine, Shandong University, Jinan, Shandong, China; ^2^ National Health Council (NHC) Key Lab of Health Economics and Policy Research, Shandong University, Jinan, Shandong, China; ^3^ China Center for Health Development Studies, Peking University, Beijing, China; ^4^ Jinan Municipal Health Commission, Jinan, Shandong, China

**Keywords:** cervical intraepithelial neoplasia, married women, risk factors, cervical cancer, low socioeconomic status

## Abstract

**Objectives:**

This study aimed to investigate the prevalence of cervical lesions in married women with low socioeconomic status, and the related risk factors to provide evidence for the development of cervical cancer prevention strategies.

**Methods:**

Descriptive analysis was employed to estimate the prevalence of cervical lesions. Univariate analysis and binary logistic regression were used to investigate the association between the related variables and cervical intraepithelial neoplasia (CIN).

**Results:**

Among 142,677 women aged 35–64 years, 787 (0.55%) cervical lesions were detected. Being in the age group of 35–44 years, high or technical secondary school level education, living at higher level regions of economic development, and abnormal leucorrhea were associated with increased risk of cervical lesions. Menopause was found to be protective.

**Conclusion:**

Married women with lower socioeconomic status had a lower prevalence of cervical lesions but had a higher prevalence of cervical cancer. Those aged 35–44 years, with high or technical secondary school level education, living at higher level regions of economic development, with abnormal leucorrhea, and who were pre-menopausal were at higher risk for cervical lesions.

## Introduction

Cervical cancer is the fourth most common cancer and the fourth primary cause of cancer-related deaths in women, with an estimated 604,000 new cases and 342,000 deaths worldwide in 2020 [1]. The global incidence of cervical cancer is 6.5% in women. In China, cervical cancer ranks sixth among female cancers, with an incidence rate of 11.0 per 100,000 people, accounting for 18.2% of global cervical cancer cases and 5.2% of Chinese cancer cases [2].

The risk factors of cervical lesions in rural women have received much attention at home and abroad, but there are significant differences in the results of studies from varying regions and socioeconomic statuses. Most studies worldwide [3–6] demonstrated that rural women with lower levels of education and urban women with lower income have decreased awareness about lower cervical cancer awareness and screening rates. And their decreased awareness regarding risky sexual behavior, including not using such as using contraception, can accelerate the development of cervical lesions.

To help such vulnerable groups, China offered free cervical cancer screening to rural women in 2009 and expanded screening to rural and urban subsistence (i.e., urban women with non-agricultural household registration whose *per capita* household income is below the local minimum standard of living) women in 2020. Following WHO recommendations (WHO, 2007) and considering the demography characteristics and the financial situation in China, the important target age for cervical cancer screening in China is defined as 35–64 years old [7]. This has also been confirmed in other studies [8–10]. In China, the screening rate for cervical cancer is very low in women aged 35–64 years [11]. These women have a high incidence of cervical lesions, including cervical cancer; therefore, screening for cervical cancer is necessary. The detection and correction of risk factors in this age group will aid in the prevention of cervical cancer.

In a study by Zhao et al [12], using data from the National Cervical Cancer Screening Program in 2021 in rural China, showed that the early detection rate increased from 89.60% (11,883) in 2012 to 92.80% (26,962) in 2018 *P* < 0.001). Jinan is the capital city of Shandong Province in China, with a permanent population of 8.91 million and about 500,000 rural women and urban subsistence women in need of cervical cancer screening. Therefore, there is an urgent need to understand the risk factors associated with cervical lesions in women with low socioeconomic status.

This study comprised rural and urban subsistence women in Jinan, China, with low socioeconomic status. All rural and urban subsistence women were accepted for cervical cancer screening. All participants were offered ThinPrep cytology tests (TCT) and HPV tests to evaluate the prevalence of cervical lesions, estimate risk factors of cervical lesions, make practical suggestions on risk factors, and provide evidence for nationwide prevention strategies for cervical cancer programs.

## Methods

### Participants and Study Design

We included 197972 rural women and urban women on subsistence allowance in the database of the Jinan 2020 Cervical Cancer Screening Program. This program was jointly launched by the Jinan Municipal Health Commission and Women’s Joint Committee and was conducted simultaneously by 95 screening agencies in 13 administrative districts of the city. According to the WHO recommendations and the current cervical cancer screening policy in China, only women aged 35–64 years were included in this screening program and study. In addition, a total of 55,295 cases with missing independent variables (education level, ethnic group, economic development level of place of residence, family history of cancer, number of pregnancies, cleanliness, leucorrhea abnormalities, contraception, and menopause) were excluded to avoid bias in association analyses. Accordingly, 142,677 were finally included in the study.

### Measurements Outcomes

The first step was to perform TCT analysis, and the results were divided into no lesions and lesions or suspected lesions and no lesions. In the second step, HPV DNA was detected, and the results were divided into HPV16/18 positive cases and others. In the third step, the cases with lesions or suspected lesions in TCT examination and HPV16/18 positive cases in the HPV DNA test were tested by colposcopy, and the cases were graded. The specific detection and determination steps are as follows:

TCT analysis was performed at the transitional zone between the old and new squamous-columnar epithelium; the results were classified as follows: a) normal; b) atypical squamous cells of undetermined significance; c) low-grade squamous intraepithelial lesion; d) atypical squamous cells that could not be ruled out from being high-grade squamous intraepithelial lesions (HSILs); and e) HSIL. Simultaneously, HPV DNA testing was conducted using a polymerase chain reaction test, and HPV type was analyzed. If the test result was high-risk HPV of subtype 16/18, colposcopy was performed together with the abnormal cases in the TCT examination.

Colposcopy revealed microscopic details of the cervical epidermal lesions. Simultaneously, during colposcopy, several pieces of tissue are clipped from the suspicious site for histological diagnosis to determine the cervical cancer or grade of cervical lesions. Finally, the results were stratified into the normal, CIN I, II, III, and cervical cancer groups. CIN I were defined as low-grade cervical intraepithelial neoplasia, and CIN II/III was defined as high-grade cervical intraepithelial neoplasia, which is precancerous. Cervical cancer progresses into cancer. In the analysis of influencing factors of cervical lesions, CIN I, CIN II/III, and cervical cancer were defined as abnormal cervical lesions. The final dependent variable was divided into two groups: the cervical lesion group and the normal group.

### Independent Variables

Patients were stratified into the 35–44-year-old, 45–54-year-old, and 55–64-year-old groups. Educational level was separated into four categories: primary school or below, junior high school, senior high school or technical secondary school, and college or above. According to the gross domestic product (GDP) *per capita* of districts and counties in Jinan in 2020, regions with a GDP *per capita* of >20,000 yuan were considered areas of high economic development; regions with a GDP *per capita* of 10,000–20,000 yuan were areas of medium levels of economic development, and regions with a GDP *per capita* of lower than 10,000 yuan were areas of low levels of economic development. Vaginal cleanliness level was assessed using the microscopic observation of vaginal discharge to determine the presence of inflammatory infections in the vagina by the number of *Bacillus* vaginalis, other bacteria, epithelial cells, and white blood cells in discharge. Clinically, a vaginal cleanliness level of I or II is normal. Ethnic groups were divided into Han and others. The number of parturitions was divided into three categories: no parturition, one to two experiences, and three or more experiences. We determined whether participants had a family history of cervical cancer and used contraception. The family history of cervical cancer refers to first-degree female relatives (mother and second-degree relatives, grandmother, aunt, etc.) with cervical cancer. Contraception includes condoms, birth control pills, intrauterine devices (IUDs), and other forms of effective contraception. Menopausal conditions were divided into pre-menopausal or postmenopausal groups.

### Statistical Analysis

Initial analysis for the association between cervical lesions with each variable was performed using the Chi-square test. Fisher’s exact test was used for small samples. Univariate logistic regression was used to explore the relationship between independent variables and cervical lesions. Odds ratio (OR) and 95% confidence intervals (CI) were evaluated. Statistical significance was set at *P* < 0.05 All statistical analyses were conducted using software (Stata, version 17.0; StataCorp, College Station, TX, USA).

## Results

### Participants Characteristics

Table 1 illustrate that, a total of 142,677 married women aged 35–64 years in rural and urban areas of Jinan were included. The mean age of participants was 50.11 (standard deviation, 7.58) years; 39,196 (27.47%) lived in areas with lower economic development levels, 59,520 (41.72%) had a primary school education or lower, and 141,970 (99.5%) were Han Chinese. Moreover, 5,798 (4.06%) of participants reported a family history of cervical cancer, only 344 (0.24%) had no pregnancy experience, 127,789 (89.57%) had normal vaginal cleanliness levels (I or II), 2,497 (1.75%) had abnormal leucorrhea, 71,861 (50.37%) had no contraceptive use, and 81,065 (56.82%) were postmenopausal.

**TABLE 1:** Characteristics of participants (Jinan, China. 2021).

Variables	Number	Percentage
Observations	142,677	100
Age group		
35–44	36,359	25.48
45–54	61,246	42.93
55–64	45,072	31.59
Education level		
Primary or below	59,520	41.72
Middle school	65,190	45.69
High or technical secondary school	11,480	8.04
College school or above	6,487	4.55
Ethnic group		
Han	141,970	99.50
Minority ethnic	707	0.50
Regional economic development level^a^		
Lower	39,196	27.47
Medium	28,009	19.63
Higher	75,472	52.90
Family History of Cervical Cancer		
No	136,879	95.94
Yes	5,798	4.06
The number of pregnancies		
0	344	0.24
1 or 2	105,234	73.76
3 or above	37,099	26.00
Vaginal cleanliness level		
I or II	127,789	89.57
III or IV	14,888	10.43
Leucorrhea abnormality		
No	140,180	98.25
Yes	2,497	1.75
Contraceptive use		
No	71,861	50.37
Yes	70,816	49.63
Menopausal status		
Premenopausal	61,612	43.18
Postmenopausal	81,065	56.82

^a^ Regional economic development level describes the macro-level socioeconomic status of the geographic region where the women reside (e.g., GDP *per capita*, healthcare infrastructure).

### Prevalence of Cervical Lesions

Table 2 illustrate that, a total of 787 women (0.55%) were diagnosed with cervical lesions-503 (0.35%) had CIN I, 254 (0.18%) had CIN II/III, and 30 (0.02%) had invasive cervical cancer. The Chi-square test showed that there were significant differences between women with cervical lesions (n = 787) and those with normal histology (n = 141,890) in aspects of age, education level, economic development level of residence, number of abortions, contraceptive use, abnormal leucorrhea, and menopausal status (*P* < 0.05). However, there seemed to be no significant difference between the aforementioned populations in aspects of ethnic group, family history of cancer, number of parturition, and vaginal cleanliness level. See Table 3.

**TABLE 2:** Correlation between general demographic characteristics and cervical lesions in participants (Jinan, China. 2021).

Variables	Normal (%)	CIN I (%)	CIN II/III (%)	Cervical cancer (%)	Cervical lesion (%)
Observations	141,890 (99.45)	503 (0.35)	254 (0.18)	30 (0.02)	787 (0.55)
Age group					
35–44	36,097 (99.28)	158 (0.44)	100 (0.28)	4 (0.01)	262 (0.72)
45–54	61,246 (99.43)	236 (0.39)	97 (0.16)	17 (0.03)	350 (0.57)
55–64	44,897 (99.61)	109 (0.24)	57 (0.13)	9 (0.02)	175 (0.39)
χ^2^		24.658	27.429		41.289
*P* value		<0.001	<0.001	0.233^a^	<0.001
Education level					
Primary or below	59,267 (99.57)	168 (0.28)	73 (0.12)	12 (0.02)	253 (0.43)
Middle school	64,793 (99.39)	250 (0.38)	133 (0.20)	14 (0.02)	397 (0.61)
High or technical secondary school	11,388 (99.20)	59 (0.52)	33 (0.29)	0 (0)	92 (0.80)
College school or above	6,442 (99.31)	26 (0.40)	15 (0.23)	4 (0.06)	45 (0.69)
χ^2^		19.190	21.624		36.732
*P* value		<0.001	<0.001	0.065^a^	<0.001
Ethnic group					
Han	141,189 (99.45)	499 (0.35)	253 (0.18)	29 (0.02)	781 (0.55)
Minority ethnic	701 (99.15)	4 (0.57)	1 (0.14)	1 (0.14)	6 (0.85)
χ^2^					1.143
*P* value		0.322^a^	1.000^a^	0.138^a^	0.285
Regional economic development level					
Lower	39,028 (99.57)	105 (0.27)	51 (0.13)	12 (0.03)	168 (0.43)
Medium	27,844 (99.41)	93 (0.33)	67 (0.24)	5 (0.02)	165 (0.59)
Higher	75,018 (99.40)	305 (0.40)	136 (0.18)	13 (0.02)	454 (0.60)
χ^2^		14.062	11.000		14.958
*P* value		0.001	0.004	0.309^a^	0.001
Family History of cervical cancer					
No	136,124 (99.45)	485 (0.36)	242 (0.18)	28 (0.02)	755 (0.55)
Yes	5,766 (99.45)	18 (0.31)	12 (0.21)	2 (0.03)	32 (0.55)
χ^2^		0.303	0.284		<0.001
*P* value		0.582	0.594	0.346^a^	0.997

^a^ Fisher’s exact test.

**TABLE 3:** Correlation between clinic characteristics and cervical lesions in participants (Jinan, China. 2021).

Variables	Normal (%)	CIN I (%)	CIN II/III (%)	Cervical cancer (%)	Cervical lesion (%)
The number of pregnancies					
0	343 (99.71)	1 (0.29)	0 (0)	0 (0)	1 (0.29)
1 or 2	104,688 (99.48)	348 (0.33)	177 (0.17)	21 (0.02)	546 (0.52)
3 or above	36,859 (99.35)	154 (0.42)	77 (0.21)	9 (0.02)	240 (0.65)
χ^2^					
*P* value		0.055^a^	0.272^a^	0.700^a^	0.015^a^
Vaginal cleanliness level					
I or II	127,079 (99.44)	459 (0.36)	227 (0.18)	24 (0.02)	710 (0.56)
III or IV	14,811 (99.48)	44 (0.30)	27 (0.18)	6 (0.04)	77 (0.52)
χ^2^		1.534	0.010	2.933	0.359
*P* value		0.216	0.921	0.087	0.549
Leucorrhea abnormality					
No	139,418 (99.46)	485 (0.35)	249 (0.18)	28 (0.02)	762 (0.54)
Yes	2,472 (99.00)	18 (0.72)	5 (0.20)	2 (0.08)	25 (1.00)
χ^2^		9.838	0.076		9.366
*P* value		0.002	0.783	0.096^a^	0.002
Contraceptive use					
No	71,431 (99.40)	238 (0.34)	102 (0.14)	17 (0.02)	430 (0.60)
Yes	70,459 (99.50)	265 (0.37)	152 (0.21)	13 (0.02)	357 (0.50)
χ^2^		1.099	9.154	0.589	5.776
*P* value		0.294	0.002	0.443	0.016
Climacteric status					
Premenopausal	61,183 (99.30)	229 (0.28)	111 (0.14)	18 (0.02)	429 (0.70)
Postmenopausal	80,707 (99.56)	274 (0.45)	143 (0.23)	12 (0.02)	358 (0.44)
χ^2^		26.335	17.953	0.119	41.389
*P* value		<0.001	<0.001	0.730	<0.001

a Fisher’s exact test.

**FIGURE 1:**
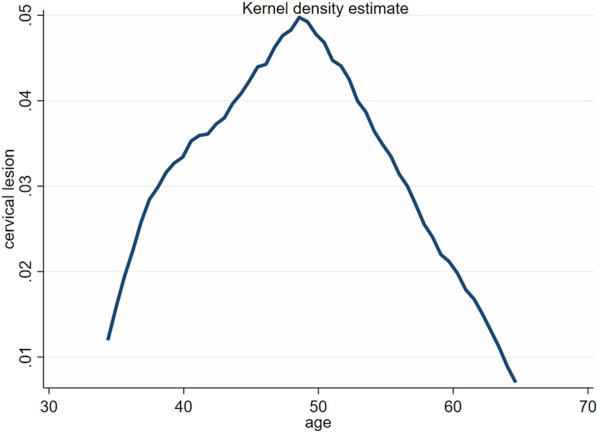
The age distribution of cervical lesions (Jinan, China. 2021).

Figure 1 illustrate that, in the kernel density curve, the number of cervical lesions was mostly concentrated in the age group of 35–54 years old. At the same time, the waveform shifted to the left and the horizontal width decreased, indicating that the nuclear density of the number of patients with cervical lesions tended to move in the direction of age.

### Logistic Regression Analysis of the Relationship Between Related Variables and Cervical Lesions

Univariate logistic regression analysis showed that the risk factors of cervical lesion were an age between 34 and 44 years (OR = 1.33, 95% CI: 1.03–1.72) and between 45 and 54 years (OR = 1.29, 95% CI: 1.05–1.59), middle school (OR = 1.21, 95% CI: 1.02–1.43) and high or technical secondary school (OR = 1.51, 95% CI: 1.16–1.95) education level, living in a region of medium (OR = 1.31, 95% CI: 1.05–1.63) and high level of economic development (OR = 1.40, 95% CI: 1.16–1.69). The relevant protective factor was postmenopausal (OR = 0.78, 95% CI: 0.65–0.94). Contraceptive use was not an independent risk factor for cervical lesions. See Table 4.

**TABLE 4:** Risk factors for cervical lesions assessed by univariate logistic regression (Jinan, China. 2021).

Variables	OR	95%CI	*P* value
Age group			
55–64	Ref		
45–54	1.29	1.05–1.59	0.016
35–44	1.33	1.03–1.72	0.028
Education level			
Primary or below	Ref		
Middle school	1.21	1.02–1.43	0.030
High or technical secondary school	1.51	1.16–1.95	0.002
College school or above	1.20	0.86–1.69	0.281
Ethnic group			
Han	Ref		
Minority ethnic	0.68	0.30–1.53	0.353
Regional economic development level			
Lower	Ref		
Medium	1.31	1.05–1.63	0.017
Higher	1.40	1.16–1.69	<0.001
Family cancer			
No	Ref		
Yes	0.94	0.66–1.34	0.725
The times of pregnancy			
0	Ref		
1–2	1.55	0.22–11.09	0.661
3 or above	1.83	0.26–13.12	0.547
Vaginal cleanliness level			
Ⅰ or Ⅱ	Ref		
Ⅲ or Ⅳ	0.83	0.66–1.06	0.135
Leucorrhea abnormality			
No	Ref		
Yes	1.64	1.10–2.46	0.016
Contraception			
No	Ref		
Yes	1.04	0.89–1.21	0.647
Menopause			
No	Ref		
Yes	0.78	0.65–0.94	0.009

Abbreviations: OR, odds ratio; CI, confidence interval.

## Discussion

### Principal Findings

The study included 142,677 participants with an overall cervical lesion prevalence of 0.55%—a rate lower than reported in comparable international and Chinese studies [13–18]. Notably, the cervical cancer prevalence of 0.02% (20 per 100,000) exceeded both the WHO global estimate (13.3/100,000) and China’s national estimate (15.6/100,000) for 2020 [19]. Key risk factors identified include age 35–44 years, high/technical secondary education, residence in economically developed regions, abnormal leucorrhea, and premenopausal status. These findings underscore the need for targeted cervical cancer screening and preventive education campaigns in these subpopulations. Our age-stratified analysis (35–64 years) aligns with established screening protocols and other studies [20–22].

The study found that elevated cervical cancer risk among middle-aged women aligned with global epidemiological trends, reflecting the interplay of behavioral exposures and biological vulnerability during peak reproductive years. The elevated cervical lesion risk among women aged 35–44 and 45–54 years corresponds with global epidemiological patterns. This age window coincides with peak sexual activity, where behavioral risks (e.g., multiple partners, inadequate protection) may intersect with biological susceptibility. Supporting evidence includes a southern Ethiopian study showing maximal precancerous lesion detection at 30–39 years [23], and Chinese data identifying 35–44 years as the highest-risk cohort [24]. While some studies suggest age-progressive risk patterns [25, 26], our findings concur with Beijing-based research demonstrating concentrated risk factors in women under 50 [27].

Notably, postmenopausal women (55–64 years) exhibited reduced cervical lesion risk, potentially attributable to hypoestrogenic physiological changes impacting cervical microenvironment stability [28]. This aligns with existing literature associating menopause with diminished lesion risk [29], though the clinical implications of reduced postmenopausal screening adherence require rigorous evaluation.

Contrasting global trends, our education analysis revealed a U-shaped risk pattern: secondary/high school education posed elevated cervical lesion risks relative to primary education, while college education served as a protective factor. We hypothesize this relates to differential sexual health literacy across educational stages. Women with primary education often reside in traditional environments with restricted sexual health exposure, potentially limiting high-risk behavior initiation. Secondary education coincides with sexual debut and adolescent development when curiosity about sexual activity escalates, yet formal sex education programs in these settings may inadequately address risk mitigation strategies [30]. This knowledge gap in safer sexual practices—documented cross-culturally [31]—could increase exposure to HPV transmission risks. Conversely, college-educated women typically possess enhanced health literacy and access to preventive resources, consistent with global studies identifying higher education as protective [32–34]. Additionally, lower-educated women’s reduced screening participation—a well-documented barrier [35, 36]—may artifactually depress detection rates in this group.

In addition, the study found that living in economically developed regions also emerged as a risk factor, which is essentially consistent with the “poverty paradox,” which manifests in tumor epidemiology as a greater health gap where the convergence of biological susceptibility and structural inequities in developed regions exacerbates health disparities compared to less-developed settings [37]. The elevated cervical cancer risk among socioeconomically disadvantaged women in economically developed regions stems from multifaceted systemic inequities. Disparities in healthcare access—including lower cervical screening uptake due to financial constraints, informational gaps, and HPV vaccine underutilization—intersect with behavioral vulnerabilities like limited sexual health literacy and high-risk sexual practices [38]. Chronic stress from economic precarity may impair immune responses, while overcrowded living conditions and occupational carcinogen exposure amplify biological susceptibility. Structural barriers in healthcare systems, such as implicit bias in clinical referrals and inadequate follow-up mechanisms for mobile populations, further entrench disparities [39]. These findings necessitate prioritizing intersectional policy reforms and community-empowered healthcare delivery to dismantle systemic barriers, ensuring equitable access to HPV vaccination, culturally competent screening, and stress-alleviation resources for marginalized women in high-income settings [40].

The strong association between abnormal leucorrhea and cervical lesions emphasizes clinical vigilance for this symptom. Clinicians must contextualize this symptom within intersecting vulnerabilities—low health literacy, gender norms deterring gynecological care-seeking, and economic barriers delaying diagnostic follow-up. Public health systems should combat structural iatrogenesis by integrating symptom education into community health worker programs. At the same time, community health workers should also educate women with low socioeconomic status about the symptoms of cervical cancer via health promotion initiatives and community engagement, so as to ensure that both doctors and patients can have a clear understanding of the risk factors or precursors that may lead to cervical cancer [41, 42].

### Limitations

This study has a few limitations. First, the authors were not provided with more in-depth information on lifestyle habits, including smoking, number of sexual partners, and other lifestyle factors, which may promote the occurrence of cervical lesions. Second, cervical cytology is more challenging in postmenopausal women due to their hypoestrogenic state. There was no specific screening method for postmenopausal women in this study, which may lead to biased results.

### Conclusion

Married women of lower socioeconomic status had a lower prevalence of cervical lesions but a higher prevalence of cervical cancer. The study brings to light the significant risk factors of cervical lesions, including age between 35 and 44 years, living at higher level regions of economic development, low educational status, and abnormal leucorrhea. Particular efforts should be invested towards cervical cancer prevention policy, which has to emphasize the aforementioned risk factors. Moreover, expanding cervical cancer screening coverage is also important because more women at risk of cervical lesions can be treated early, and cervical cancer can be prevented to a greater extent.

## Data Availability

All data that support the findings of this study are included in this article and its supplementary information files, further inquiries can be directed to the corresponding author.
